# Voluntary Hospital Staff Conference in Scotland

**Published:** 1921-03-05

**Authors:** 


### Voluntary Hospital Staff Conference in Scotland.

A conference, similar to that called recently by the British Medical Association at Cambridge, was held in Edinburgh last month and attended by representatives of the honorary medical staffs of voluntary hospitals in Scotland. All the teaching hospitals were represented, as well as a large number of the smaller general and special hospitals throughout the country. The meeting expressed itself as in favour of the present methods of administration of the voluntary hospitals being continued, and another important resolution was that it is not desirable that voluntary hospitals should be subsidised by the local rating authorities, except in so far as payment is made for the examination and the care of patients for whom these authorities are responsible.

Further resolutions were passed advocating increased support of hospitals by employers and employees, and contributions by non-necessitous patients, a percentage of these payments to be placed at the disposal of the honorary medical staff of the hospital where such payments are made.

A suggestion that hospitals should be advised to apply for a Government grant for nurse-training, as a branch of secondary education, is to receive further consideration.

